# The impact of folate pathway variants on the outcome of methotrexate therapy in rheumatoid arthritis patients

**DOI:** 10.1007/s10067-024-06892-w

**Published:** 2024-02-05

**Authors:** Azhar M. Nomair, Abeer Abdelati, Fatma I. Dwedar, Rehab Elnemr, Yasmine N. Kamel, Hanan M. Nomeir

**Affiliations:** 1 Department of Chemical Pathology, Medical Research Institute, Alexandria University, Alexandria, Egypt; 2 Department of Internal Medicine, Rheumatology and Clinical Immunology Unit, Faculty of Medicine, Alexandria University, Alexandria, Egypt; 3 Department of Medical Biochemistry, Faculty of Medicine, Alexandria University, Alexandria, Egypt; 4 Department of Physical Medicine Rheumatology and Rehabilitation, Faculty of Medicine, Alexandria University, Alexandria, Egypt

**Keywords:** ATIC, Methotrexate, RFC-1, Rheumatoid arthritis, TYMS, Variant

## Abstract

**Background:**

There are currently no validated criteria that entirely explain or predict response to methotrexate (MTX) treatment in rheumatoid arthritis (RA). We tried to identify the connection between three variants (*RFC1* G80A (rs1051266), *TYMS* 2R/3R (rs34743033), and *ATIC* C347G (rs2372536)) in the folate pathway of MTX metabolism and the response to MTX monotherapy in a cohort of RA cases.

**Methods:**

A prospective study on 100 RA patients on MTX monotherapy was performed. Disease activity was measured at the start of treatment and 6 months after treatment with MTX. The patients were then split into two groups: those who responded to the treatment and those who did not. The molecular genetic study for the *RFC1 (G80A)* variant was employed via the PCR-restriction fragment length polymorphism (PCR–RFLP) technique, the *ATIC (C347G)* variant was performed using TaqMan allelic discrimination real-time PCR, and the tandem repeat sequences of *TYMS (2R/3R)* were amplified by conventional PCR and detected by agarose gel electrophoresis.

**Results:**

The genotype distribution of *RFC-1 (G80A)* showed significant variations among non-responders and responders in the recessive genetic model. A significant difference was found in *TYMS (2R/3R)* in the dominant and heterozygous genetic models. However, *ATIC* (C347G) genotype frequency did not exhibit substantial link with drug response in all genetic models. Furthermore, the genotype and allele rates of the analyzed variants did not show any significant association with adverse events in all genetic models.

**Conclusion:**

The 80AA genotype of *RFC-1 G80A* and the 2R/3R or 3R/3R genotypes of *TYMS 2R/3R* are more vulnerable to the good consequences of MTX therapy.

**Table:** 

**Key Points**
• *Current recommendations support the gold standard role of MTX as a first-line monotherapy for RA patients. However, up to 40% of RA patients do not respond or exhibit partial response to MTX.* • *Persistent disease activity due to treatment unresponsiveness will affect the long-term outcomes in RA patients.* • *We aimed, through molecular genetic study, to identify the connection between three variants in the folate pathway of MTX metabolism and the response to methotrexate monotherapy in a cohort of RA patients.*

## Introduction

Rheumatoid arthritis (RA) is a female-predominant autoimmune inflammatory illness. Its prevalence varies significantly between countries. It accounts for 0.5 to 1% of the population. RA predominantly affects the synovial joint lining, leading to joint pain and swelling, which results in joint deformity, physical disability, and ultimately a lower quality of life [1]. The major treatment goal after the diagnosis is to monitor disease activity and slow the rate of joint destruction while also decreasing pain, stiffness, inflammation, and possible complications [2]. To achieve complete hindering of the disease’s progress and clinical remission, rheumatologists must monitor the progression of the disease on a frequent and accurate basis and alter therapeutic regimens as warranted. A change in the disease activity score of 28 joints (DAS28) is a standard way to assess the therapy response [3].

Disease-modifying anti-rheumatic drugs (DMARDs) have received a lot of attention during the last two decades because they can effectively stop disease progress, delay or prevent joint destruction, and substantially improve the long-term functional outcome [4].

Methotrexate (MTX) (4-amino-10-methyl folic acid) is a highly effective conventional synthetic disease-modifying anti-rheumatic drug (csDMARD) that is widely employed in RA therapy, either alone or in combination with other DMARDs [5, 6]. It is a folic acid structural analogue with potent anti-inflammatory and anti-proliferative properties. Following its administration and absorption, MTX enters the cell through reduced folate carrier-1 (RFC1), which is encoded via the human solute carrier family 19, member 1 (*SLC19A1*), or *RFC1* gene on chromosome 21. It was reported that genetic variants in the *RFC1* gene influenced RFC1 function, affecting MTX transport [1]. Within the cell, MTX is transformed into methotrexate polyglutamates (MTX-PG), which direct the long-term suppression of the folate pathway and provide single-carbon moieties required for purine and pyrimidine biosynthesis [7]. They inhibit important intracellular enzymes within the folate pathway involving dihydrofolate reductase (DHFR), thymidylate synthetase (TS), and 5-aminoimidazole-4-carboxamide ribonucleotide (AICAR) transformylase (ATIC) [8].

Thymidylate synthase is an enzyme that transforms deoxyuridylate into deoxythymidylate, which is needed for DNA synthesis and repair [9]. It is hindered by MTX-PG, thus participating in MTX anti-inflammatory and anti-proliferative properties [1]. Some variants in the *TYMS* gene, which encodes TS, look to be linked to MTX responsiveness. A genetic variant in the enhancer region (TSER) is *TYMS-TSER*-2R/3R, which comprises two or three 28-bp tandem repeats. The inclusion of a triple rather than a double 28-bp repeat has been demonstrated to enhance *TYMS* expression and might necessitate a larger MTX dosage to produce an effective therapeutic outcome; however, the potential implications of this variant on enzyme activity are still debated [10].

The ATIC enzyme changes AICAR into formyl-AICAR, which is then implicated in the de novo production of purine. Inhibition of ATIC by MTX leads to an intracellular piling up of AICAR, causing the emission of adenosine into the extracellular space. The adenosine released elevates cyclic adenosine monophosphate (cAMP), which prevents the release of pro-inflammatory cytokines that serve critical functions in the inflammatory process in RA [1].

Although MTX is the mainstay of RA treatment, toxicity remains a concern. A total of 30 to 45% of RA patients who take MTX have side effects, and 16% stop taking it because of side effects like hepatotoxicity, interstitial pneumonia, renal failure, pancytopenia, gastrointestinal dysfunction, and elevated susceptibility to infection [11].

The causes of individual non-responsiveness and the existence of adverse reactions are still hard to know. Genetic markers have recently received a lot of attention as potential indicators of MTX efficacy and toxicity. The goal of this research is to assess the relationship among *RFC1* G80A (rs1051266), *TYMS-*2R/3R (rs34743033), and *ATIC* C347G (rs2372536) variants found in the MTX metabolic cascade and the outcome (response to/or toxicity of) methotrexate therapy in Egyptian patients with RA.

## Subjects and methods

### Study population

This study is a prospective cohort experiment employed from July 2022 to April 2023. It was carried out directly after the approval of the Ethical Committee of the Faculty of Medicine, Alexandria University (approval no. 0305722, IRB no. 00012098). Informed consent was received from all people involved in the study. A hundred early rheumatoid arthritis (symptom duration ≤ 2 years) or newly diagnosed cases were consecutively gathered from the rheumatology outpatient clinic and inpatient unit in the Rheumatology Unit, Alexandria Main University Hospital. The detection of RA was based on the 2010 American College of Rheumatology (ACR)/European League Against Rheumatism (EULAR) classification criteria for RA [12]. The inclusion criteria included DMARD-naïve patients with active disease (DAS28-ESR > 3.2) [13], and only cases treated with MTX as csDMARD monotherapy were selected. Other additional medications included oral prednisolone in a dose of ≤ 10 mg/day or its equivalent (with a tapering regimen), non-steroidal anti-inflammatory drugs (NSAIDs), and folic acid, which were allowed to be taken during the study period. The exclusion criteria included patients with MTX intolerance, patients receiving csDMARDs other than methotrexate, as well as biologic (bDMARD) and targeted synthetic DMARDs (tsDMARDs).

The patients were assessed at baseline, after 3 months, and after 6 months post therapy with methotrexate. Starting doses ranged between 12.5 and 25 mg/week, regardless of the route of administration. During follow-up visits, patients who developed any of the adverse effects of MTX have been asked to reduce the dose of MTX to the lowest tolerated dose, which was 7.5 mg/week in our cohort. Then, they were categorized into a responders’ group (41 patients) and a group of non-responders (59 patients) according to the reassessment at the follow-up visit after 6 months. The non-responders are those who remained on MTX for 6 months (the study peroid) but did not exhibit enough improvement in line with EULAR response criteria (DAS28 improvement of ≤ 0.6 from baseline score), whereas the responders are those who showed improvement in their DAS28 ≥ 0.6 from baseline assessment [14]. Patients who had discontinued the MTX before completing 6 months due to MTX intolerance, adverse events, inefficacy, or required bDMARD or tsDMARDs have been excluded from the study.

Furthermore, the subjects were divided into cases with adverse events (*n* = 52) and cases without adverse events (*n* = 48) according to the development of any of the known MTX adverse effects.

### Methods

All of the following were accomplished for all of the patients who were enrolled in the study: an entire history taking with stress on the onset of the disease; a history of smoking, periodontitis, and joint symptoms (pain, tenderness, swelling, morning stiffness, and difficulty in movement); and a physical examination with stress on the number of tender and/or swollen joints, limitations of motion, deformities, rheumatoid nodules, extra-articular manifestations, and the presence, if any, of a low-grade fever and other constitutional manifestations. Disease activity estimations in the form of DAS28-ESR (scale 0–9), functional assessment by using the health assessment questionnaire (HAQ) (score range 0–3), and imaging tests for joints in the form of an X-ray or ultrasound were done on any patient if required.

### Laboratory investigations

Rheumatoid factor (RF) and anti-cyclic citrullinated protein antibody (Anti-CCP) have been requested for all participants. Routine laboratory tests including complete blood count (CBC), serum alanine aminotransferase (ALT), aspartate aminotransferase (AST), urea, creatinine, estimated glomerular filtration rate (eGFR), and inflammatory markers involving erythrocyte sedimentation rate (ESR) and C-reactive protein (CRP) were done for all cases at baseline (time of recruitment), after 3 months (follow-up visit), and after 6 months of treatment with MTX (end of study).

### Molecular genetic studies

#### DNA extraction

Whole venous blood specimens were gathered on Vacutainer ethylenediaminetetraacetic acid (EDTA) blood collection tubes from all patients. Genomic DNA extraction by column-based extraction kits (QIAamp DNA Blood Mini Kit, Catalogue number: 51104) was performed depending on the manufacturer’s manuals. The purity and concentration of DNA were identified by the Thermo Scientific NanoDrop™ 1000A Spectrophotometer at 260 and 280 nm.

#### *RFC1* genotyping

The genotyping of *RFC1* G80A was employed via the polymerase chain reaction-restriction fragment length polymorphism (PCR–RFLP) assay. It was employed in a 25 µL reaction volume, utilizing 10 pmol forward primer (5-AGTGTCACCTTCGTCCCCTC-3′), 10 pmol reverse primer (5-CTCCCGCGTGAAGTTCTT-3′), and 12.5 µL COSMO “Hot Start” PCR RED Master Mix (Willowfort, UK). The PCR conditions were performed as previously described [15]. It started with a 5-min denaturation at 95 °C, then, 35 cycles of denaturation for 15 s at 95 °C, annealing at 60 °C for 1 min, extension for 30 s at 72 °C, and a final extension at 72 °C for 7 min.

**Fig. 1:**
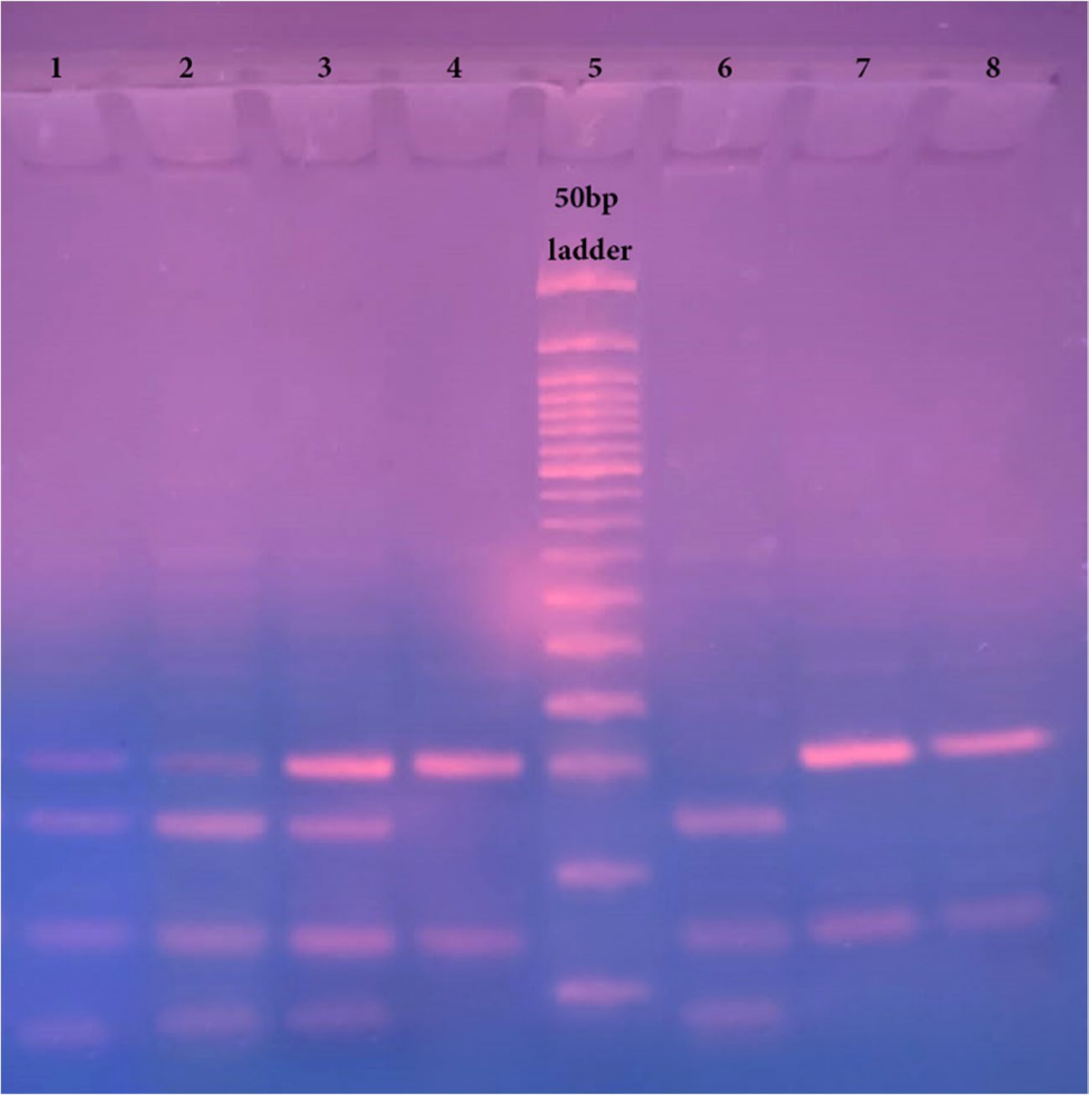
Agarose gel electrophoresis (3%) of the PCR products digested with the Fast Digest HhaI restriction enzyme. Lanes 5: 50 bp DNA Ladder (50–1000 bp); Lanes 1, 2, and 3: 80 GA heterozygote alleles (bands at 162, 125, 68, and 37 bp); Lanes 4, 7, and 8: 80 AA homozygote alleles (bands at 162 bp, and 68 bp); Lane 6: 80 GG homozygote alleles (band at 125, 68, and 37 bp)

A total of 25 µL of the PCR products (230 bp) were broken down with 1 µL of the Fast Digest HhaI enzyme (FD1854, Thermo Fisher Scientific, USA) for 5 min at 37 °C as described by the manufacturer. The digested PCR product was run on 3% agarose gel electrophoresis and stained with ethidium bromide. Individuals with the 80GG genotype had three fragments (125, 68, and 37 bp), those with the 80GA genotype had four fragments (162, 125, 68, and 37 bp), and those with the 80AA genotype had two fragments (162 and 68 bp) (Fig. 1).

#### *TYMS* genotyping

Standard PCR was used to amplify the TYMS genomic area, and the tandem repeat sequences in the 5′-end of the TYMS regulatory region [TYMS 2R/3R (rs34743033)] were detected. It was performed in a 25-μL reaction volume, via 10 pmol forward primer (5′‐GTGGCTCCTGCGTTTCCCCC‐3′), 10 pmol reverse primer (5′-GCTCCGAGCCGGCCACAGGCATGGCGCGG-3), and 12.5 µL COSMO “Hot Start” PCR RED Master Mix (Willowfort, UK). The PCR condition was performed as described previously, with modifications [16]. It comprised a 5-min initial denaturation at 94 °C, then proceeded to 35 cycles with denaturation for 1 min at 94 °C, annealing at 60 °C for 30 s, extension for 30 s at 72 °C followed by a final extension at 72 °C, for 10 min.

**Fig. 2:**
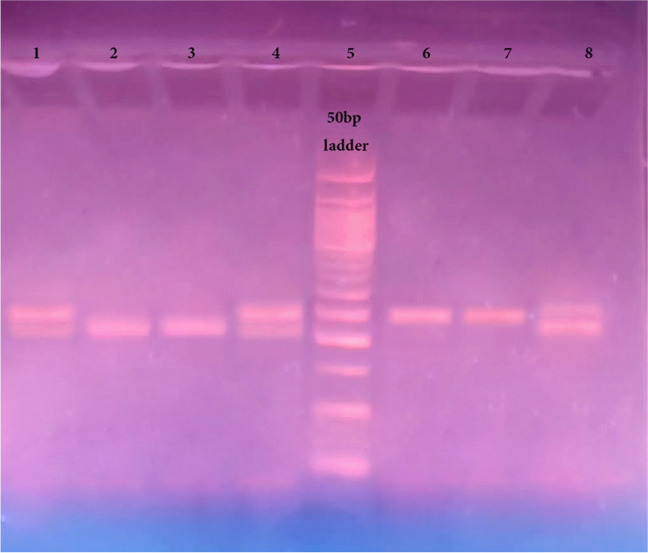
Agarose gel electrophoresis (3%) of PCR products of the amplified tandem repeat sequences in the 5′-terminal of the *TYMS* regulatory region. Lane 5: 50 bp DNA Ladder (50–1000 bp); Lanes 1, 4, and 8: heterozygotes (*TYMS* 2R3R) produced bands at 220 bp and 248 bp; Lanes 2 and 3: homozygotes of the double repeats (*TYMS* 2R2R), produced bands at 220 bp; Lanes 6 and 7: homozygotes of the triple repeats (*TYMS 3R3R*) produced bands at 248 bp

The PCR product was directly run on 3% agarose gel electrophoresis and stained with ethidium bromide. In the existence of homozygotes of the double repeat (*TYMS* 2R2R), a 220-bp fragment was created, while heterozygotes (*TYMS* 2R3R) produced two fragments of 220 and 248 bp. In the existence of homozygous *TYMS* triple repeat (*TYMS* 3R3R), a fragment with 248 bp was generated (Fig. 2).

#### *ATIC* genotyping

The *ATIC* (C347G) genotyping was done with TaqMan allelic real-time PCR and fluorogenic binding probes. A ready-made TaqMan SNP Genotyping Assay (C__16218146_10; catalogue number: 4351379) from Thermo Fisher Scientific was employed. The reaction volume was 20 µL for each sample. It was made up of 10 µL of TaqMan Genotyping Master Mix (catalogue number: 4371353; Thermo Fisher Scientific), 3.0 µL of extracted DNA, 1.0 µL of 20X SNP assay mixture (primers and probes), and 6.0 µL of nuclease-free water. The following thermal cycling circumstances were implemented: 10 min at 95 °C for enzyme activation, 40 cycles of 15 s at 95 °C for denaturation, and 1 min at 60 °C for annealing and extension. TaqMan Genotyper Software (Applied Biosystems) was used to analyze the data for genotyping, and allelic discrimination plots were constructed for each run to visualize the genotypes of the complete cohort.

### Statistical analysis

The sample size was calculated via NCSS 2004 and PASS 2000 software. It recorded 90% power to identify an effect size (W) of 0.4 in the treatment response utilizing a 2-degree-of-freedom Chi-square test with a significance level (alpha) of 0.05 considering a 10% loss follow-up rate. Data was entered into the computer and processed with the IBM SPSS software program, version 20.0. IBM Corporation, Armonk, New York. To compare the two categorical groups, the chi-square test was utilized. When greater than 20% of the cells had an anticipated enumeration of less than 5, the Fisher Exact or Monte Carlo correction test was applied. The Kolmogorov–Smirnov test was implemented to determine the normality of continuous data. For normally distributed quantitative variables, the student *t* test and paired *t* test were applied. For not-normally distributed quantitative parameters, the Mann–Whitney test and Wilcoxon signed rank tests were applied. By comparing expected and observed genotype frequencies, the agreement with the Hardy–Weinberg equilibrium (HWE) was determined. All genotype frequencies were in HWE, except for the frequency of the *RFC-1* genotype, which deviated from HWE. This is due to the overrepresentation of the heterozygous genotype frequency. Under homozygous, heterozygous, dominant, and recessive genetic models, we calculated genotype-specific odds ratios (ORs). People who were homozygous for the common allele served as referents for homozygous, heterozygous, and dominant models. We utilized those with one or two copies of the common allele as the reference group for the recessive model. The acquired findings were declared significant at the 5% level. In addition, univariate logistic regression analysis was implemented to determine the relationship between methotrexate non-response and the individual gene polymorphisms, age, gender, and other clinical factors.

## Results

### Patients’ clinical characteristics

A total of 100 cases of rheumatoid arthritis were on MTX monotherapy. According to the EULAR response criteria, they were subdivided into 41 patients as responders, and their mean age was 39.2 ± 7.9 years, and 59 patients as non-responders, and their mean age was 39.0 ± 8.7 years. Thirty-two females were present in the responders’ group, while 46 females were found in the non-responders group. The median disease duration at the time of recruitment in the non-responders group was 10.0 (3.0–24.0) month, and in the responders group was 9.0 (3.0–24.0) month, with no significant difference between the two groups (*p* = 0.251). By analyzing the relation between the patients’ characteristics and the clinical response to methotrexate, no significant differences were observed between the two groups as regards smoking (*p* = 0.646), body mass index (0.524), as well as comorbidity such as diabetes mellitus (*p* = 0.440) and hypertension (*p* = 0.13).

Baseline activity markers and disease activity were measured in all patients using the ESR, CRP, DAS 28-ESR, and HAQ score, and then they were re-measured 3 (not shown) and 6 months after MTX therapy MTX. The results showed no statistically significant difference between the two groups at the baseline assessment in ESR, CRP, DAS28-ESR (*p* = 0.067, 0.077, 0.992, respectively), whereas baseline HAQ score showed significant higher value in responder than in non-responder group (*p* = 0.016). The baseline and follow-up activity markers and disease activity score of the two studied groups are presented in Table 1

**Table 1:** The relation between markers of disease activity and clinical response to methotrexate

Disease activity markers	Non-responders (*n* = 59)	Responders (*n* = 41)	Test of Sig	*p*
Baseline ESR (1st hr) Median (Min.–Max.)	50.0 (20.0–113.0)	56.0 (22.0–110.0)	*U* = 948.0	0.067
Follow-up ESR (1st hr) Median (Min.–Max.)	36.0 (17.0–120.0)	22.0 (10.0–36.0)	*U* = 260.50	< 0.001^*^
*Z* (*P*_*1*_)	4.499 (< 0.001^*^)	5.580 (< 0.001^*^)		
Baseline CRP (mg/dl) Median (Min.–Max.)	12.0 (0.7–83.0)	14.0 (6.0–42.0)	*U* = 957.00	0.077
Follow-up CRP (mg/dl) Median (Min.–Max.)	9.2 (2.0–84.0)	5.3 (2.0–15.1)	*U* = 639.00^*^	< 0.001^*^
*Z* (*P*_*1*_)	5.199 (< 0.001^*^)	5.501 (< 0.001^*^)		
Baseline DAS 28-ESR Mean ± SD	5.1 ± 1.0	5.1 ± 0.8	*t* = 0.010	0.992
Follow-up DAS 28-ESR Mean ± SD	4.8 ± 0.9	3.3 ± 0.8	*t* = 8.637^*^	< 0.001^*^
*t*_1_ (*P*_*1*_)	5.762 (< 0.001^*^)	10.926 (< 0.001^*^)		
Baseline HAQ score Median (Min.–Max.)	1.8 (0.4–2.4)	1.9 (1.0–2.5)	*U* = 868.50^*^	0.016^*^
Follow-up HAQ score Median (Min.–Max.)	1.4 (0.4–2.2)	0.7 (0.2–1.3)	*U* = 296.50^*^	< 0.001^*^
*Z* (*P*_*1*_)	5.334 (< 0.001^*^)	5.583 (< 0.001^*^)		

*ESR* erythrocyte sedimentation rate, *CRP* C-reactive protein, *DAS 28* Disease Activity Score of 28 joints, *HAQ* Health Assessment Questionnaire, *SD* standard deviation, *t* Student *t* test, *U* Mann Whitney test, *t*_*1*_ paired *t* test, *Z* Wilcoxon signed ranks test, *p p*-value for comparing the two studied groups, *P*_*1*_* p*-value for comparing baseline disease activity and follow-up disease activity

^*^p ≤ 0.05

### Routine laboratory investigations

The comparison among the non-responders’ and responders’ groups regarding routine laboratory investigations including hemoglobin, white blood count, platelets, AST, creatinine, and eGFR revealed no significant differences between the two groups with *p* = 0.102, *p* = 0.526, *p* = 0.329, *p* = 0.131, *p* = 0.902, and* p* = 0.984, respectively. However, there was significant difference in serum urea and ALT level with *p* ≤ 0.001.

### Serology markers

Of the MTX responders, 87.8% were seropositive for RF, whereas 89.8% were positive in the non-responders’ group. The anti-CCP was positive in 85.4% of the responders’ group, while it was 91.5% positive in the non-responders’ group (Table 2).

**Table 2:** The relation between the serology markers and treatment data and the clinical response to methotrexate

	Non-responders (*n* = 59)	Responders (*n* = 41)	Test of Sig	*p*
RF				
Negative (≤ 8 IU/ml)	6 (10.2%)	5 (12.2%)	*χ*^2^ = 0.101	^FE^*p* = 0.756
Positive (˃8 IU/ml)	53 (89.8%)	36 (87.8%)		
Median (Min.–Max.)	110 (29–400)	30 (8–375)	*U* = 396.0^*^	< 0.001^*^
Anti-CCP				
Negative (≤ 18 u/ml)	5 (8.5%)	6 (14.6%)	*χ*^2^ = 0.937	^FE^*p* = 0.351
Positive (˃18u/ml)	54 (91.5%)	35 (85.4%)		
Median (Min.–Max.)	129 (36–500)	269 (23–720)	*U* = 646.0^*^	0.012^*^
Dose of MTX (mg/week)				
Median (Min.–Max.)	25 (12.5–25)	25 (12.5–25)	*U* = 1178.50	0.813
Route				
IM	34 (57.6%)	25 (61%)	*χ*^2^ = 0.251	0.882
SC	11 (18.6%)	8 (19.5%)		
Oral	14 (23.7%)	8 (19.5%)		
Adverse events				
GIT upset	18 (30.5%)	15 (36.6%)	*χ*^2^ = 0.404	0.525
Elevated liver enzymes	5 (8.5%)	2 (4.9%)	*χ*^2^ = 0.481	^FE^*p* = 0.697
Leucopenia	4 (6.8%)	0 (0%)	*χ*^2^ = 2.895	^FE^*p* = 0.142
Excessive hair fall	19 (32.2%)	14 (34.1%)	*χ*^2^ = 0.041	0.839
Patients on prednisolone				
No	2 (3.4%)	4 (9.8%)	*χ*^2^ = 1.738	^FE^*p* = 0.224
Yes	57 (96.6%)	37 (90.2%)		
Dose of prednisolone (mg/day)				
Median (Min.–Max.)	5 (5–10)	5 (2.5–10)	*U* = 705.0^*^	0.002^*^
Patients on NSAIDs	49 (83.1%)	36 (87.8%)	*χ*^2^ = 0.429	^FE^*p* = 0.513

*RF* rheumatoid factor, *anti-CCP* anti-cyclic citrullinated peptide, *MTX* methotrexate, *NSAID* nonsteroidal anti-inflammatory drugs, *SD* standard deviation, *U* Mann Whitney test, *χ*^*2*^ Chi-square test, *FE* Fisher exact, *p p*-value for comparing the two studied groups

^*^p ≤ 0.05

### Lines of treatment

The median dose of MTX given to both groups was 25 mg/week. GIT upset (36.6%) was the most frequent adverse event in the responders’ group, while excessive hair fall (32.2%) was the most frequent in the non-responders’ group. The details of the therapy given to the studied groups are represented in Table 2.

### Genotyping and allele frequency

In the non-responders’ group, the allele frequencies of *RFC-1* 80G (rs1051266) and *TYMS* 2R (rs34743033) were 45.8% and 39.8%, respectively, while the frequency of the *ATIC* G (rs2372536) allele was 32.2%. Non-statistical significance was discovered among non-responders’ and responders’ groups in the allele models among all the studied variants. The allele and genotype rates of the analyzed variants among non-responders and responders are presented in Table 3.

**Table 3:** The association between different genetic models of *ATIC*, *TYMS*, and *RFC-1* genes and the clinical response to methotrexate

	Non-responders (*n* = 59)	Responders® (*n* = 41)	*p*	OR (LL–UL 95%C. I)
*RFC-1* G80A (rs1051266)				
GG ®	3 (5.1%)	5 (12.2%)		
AA^a^	8 (13.6%)	15 (36.6%)	0.890	0.889 (0.168–4.716)
GA^b^	48 (81.4%)	21 (51.2%)	0.085	3.810 (0.833–17.426)
AA + GA^c^	56 (94.9%)	36 (87.8%)	0.211	2.593 (0.584–11.519)
GA + GG ®	51(86.4%)	26 (63.4%)		
AA^d^	8 (13.6%)	15 (36.6%)	0.009^*^	0.272 (0.102–0.724)
G allele ®	54 (45.8%)	31 (37.8%)		
A allele^e^	64 (54.2%)	51 (62.2%)	0.263	0.720 (0.405–1.280)
*TYMS* 2R/3R (rs34743033)				
2R/2R ®	14 (23.7%)	2 (4.9%)		
3R/3R ^a^	26 (44.1%)	18 (43.9%)	0.053	0.206 (0.042–1.021)
2R/3R ^b^	19 (32.2%)	21 (51.2%)	0.013*	0.129 (0.026–0.644)
3R/3R + 2R/3R^c^	45 (76.3%)	39 (95.1%)	0.022^*^	0.165 (0.035–0.771)
(2R/3R + 2R/2R) ®	33 (55.9%)	23 (56.1%)		
3R/3R ^d^	26 (44.1%)	18 (43.9%)	0.987	1.007 (0.451–2.247)
2R allele ®	47 (39.8%)	25 (30.5%)		
3R allele ^e^	71 (60.2%)	57 (69.5%)	0.177	0.663 (0.365–1.204)
*ATIC* C347G (rs2372536)				
CC ®	27 (45.8%)	25 (61%)		
GG ^a^	6 (10.2%)	5 (12.2%)	0.874	1.111 (0.301–4.100)
CG ^b^	26 (44.1%)	11 (26.8%)	0.085	2.189 (0.898–5.332)
GG + CG ^c^	32 (54.2%)	16 (39.0)	0.136	1.852 (0.824–4.163)
CG + CC ®	53 (89.8%)	36 (87.8%)		
GG ^d^	6 (10.2%)	5 (12.2%)	0.750	0.815 (0.231–2.874)
C allele ®	80 (67.8%)	61 (74.4%)		
G allele ^e^	38 (32.2%)	21 (25.6%)	0.315	1.380 (0.736–2.587)

Results are expressed in *n* (%)

*OR* odds ratio, ® reference group, *CI* confidence interval, *LL* lower limit, *UL* upper limit, *a* homozygous genetic model, *b* heterozygous genetic model, *c* dominant genetic model, *d* recessive genetic model, *e* allele model, *p p*-value for univariate regression analysis, *ATIC* 5-aminoimidazole-4-carboxamide ribonucleotide transformylase, *TYMS* thymidylate synthetase, *RFC1* reduced folate carrier-1

^*^p ≤ 0.05

**Fig. 3:**
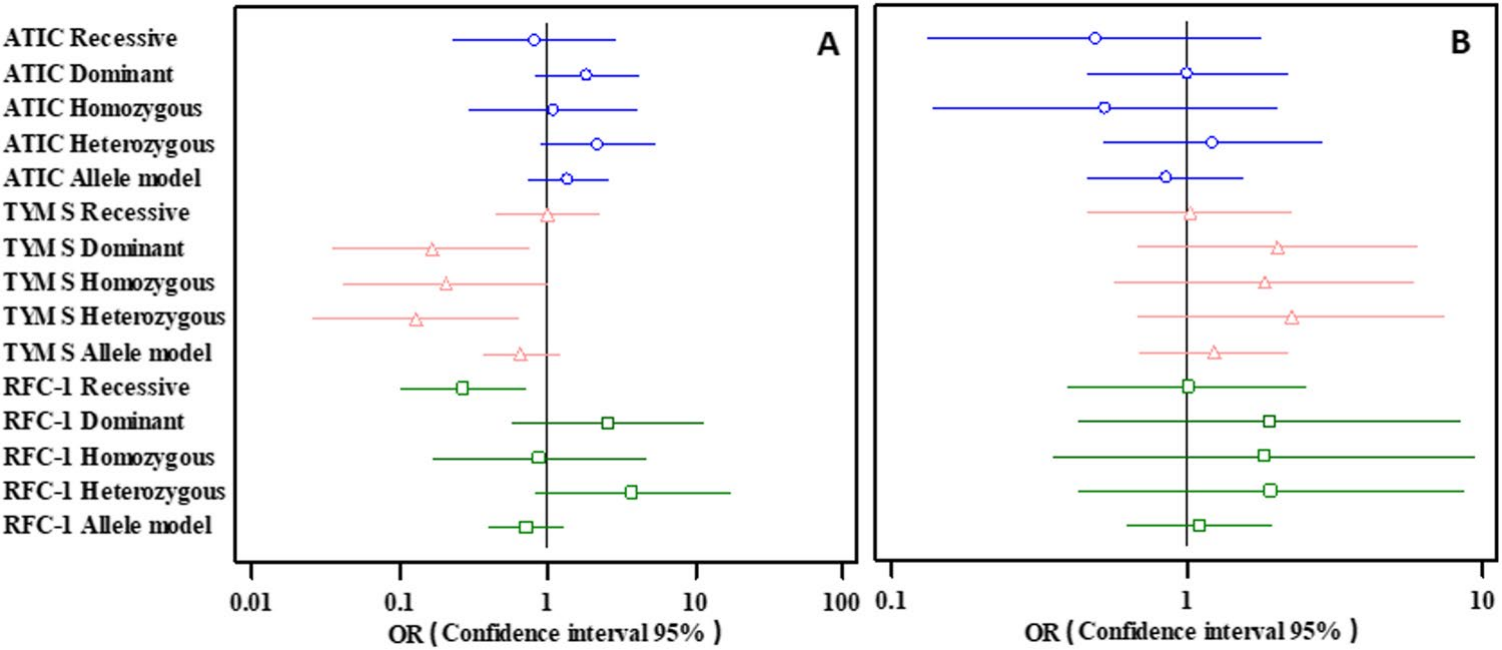
**A** Forest plot showing the association between the studied genetic variants and MTX response. **B** Forest plot showing the association between the studied genetic variants and MTX toxicity

The genotype distribution of *RFC-1* G80A (rs1051266) showed significant differences among the non-responders and responders in the recessive, *p* = 0.009, OR = 0.272 (0.102–0.724), genetic model. Also, a significant difference was found in *TYMS* 2R/3R (rs34743033) among the dominant model, *p* = 0.022, OR = 0.165 (0.035–0.771), and the heterozygous genetic model, *p* = 0.013, OR = 0.129 (0.026–0.644). On the other hand, *ATIC* C347G (rs2372536) genotype frequency did not exhibit any significant link with the drug response in all the genetic models. A forest plot showing the link between the studied genetic parameters and the MTX response is represented in Fig. 3A.

A univariate regression was implemented to figure out the role of the various clinical factors in predicting the MTX outcome. Except for TYMS (rs34743033) (2R/2R) *p* = 0.022, OR = 6.067 (1.297–28.366), and *RFC-1* (rs1051266) (GA + GG) *p* = 0.009, OR = 3.678 (1.381–9.794), none of the clinical variables retained significance in the univariate analysis. The multivariate regression analysis confirmed their role as risk factors for non-response to methotrexate (Table 4).

**Table 4:** Univariate and multivariate logistic regression analysis for the parameters affecting the non-response to methotrexate therapy

	Univariate		Multivariate	
	*p*	OR (LL–UL 95%C. I)	*p*	OR (LL–UL 95%C. I)
Age (/year)	0.877	0.996 (0.949–1.045)		
Male	0.992	1.005 (0.384–2.630)	-	
Disease duration at the time of recruitment (months)	0.267	1.053 (0.961–1.154)		
Smoking	0.348	2.909 (0.313–27.023)		
BMI (kg/m^2^)	0.468	0.968 (0.886–1.057)		
1st hr. ESR ( baseline)	0.098	0.984 (0.966–1.003)		
CRP (baseline)	0.721	0.994 (0.962–1.027)		
DAS 28-ESR (baseline)	0.992	0.998 (0.647–1.538)		
Positivity of RF-Latex	0.750	1.227 (0.348–4.325)		
Positivity of Anti-CCP	0.338	1.851 (0.525–6.532)		
MTX dose (mg/week)	0.742	1.015 (0.931–1.106)		
Positivity of prednisolone therapy	0.207	3.081 (0.537–17.679)		
ATIC (rs2372536) [GG + CG]	0.136	1.852 (0.824–4.163)		
TYMS (rs34743033) [2R/2R]	0.022^*^	6.067 (1.297–28.366)	0.022^*^	6.390 (1.304–31.325)
RFC-1 (rs1051266) [GA + GG]	0.009^*^	3.678 (1.381–9.794)	0.010^*^	3.838 (1.372–10.735)

*OR* odds ratio, *C.I* confidence interval, *LL* lower limit, *UL* upper limit

^#^All variables with *p* < 0.05 was included in the multivariate analysis

^*^p ≤ 0.05

Furthermore, the genotype and allele rates of the analyzed variants did not show any significant association with the adverse reactions in all the genetic models, as shown in Table 5. A forest plot showing the relationship between the studied genetic parameters and the MTX toxicity is represented in Fig. 3B.

**Table 5:** The association between different genetic models of *ATIC*, *TYMS*, and *RFC-1* genes and the adverse events of methotrexate

	Patients with adverse events (*n* = 52)	Patients without adverse events® (*n* = 48)	*p*	OR (LL–UL 95%C. I)
*RFC-1* G80A (rs1051266)				
GG ®	3 (5.7%)	5 (10.4%)		
AA ^a^	12 (23.1%)	11 (22.9%)	0.477	1.818 (0.350–9.455)
GA^b^	37 (71.2%)	32 (66.7%)	0.394	1.927 (0.427–8.702)
AA + GA ^c^	49 (94.2%)	43 (89.6%)	0.398	1.899 (0.429–8.417)
GA + GG ®	40 (76.9%)	37 (77.1%)		
AA ^d^	12 (23.1%)	11 (22.9%)	0.985	1.009 (0.397–2.563)
G allele ®	43 (41.3%)	42 (43.8%)		
A allele ^e^	61 (58.7%)	54 (56.2%)	0.731	1.103 (0.630–1.934)
*TYMS* 2R/3R (rs34743033)				
2R/2R®	6 (11.6%)	10 (20.8%)		
3R/3R ^a^	23 (44.2%)	21 (43.8%)	0.314	1.825 (0.565–5.895)
2R/3R ^b^	23 (44.2%)	17 (35.4%)	0.181	2.255 (0.686–7.416)
3R/3R + 2R/3R ^c^	46 (88.5%)	38 (79.2%)	0.211	2.018 (0.672–6.058)
(2R/3R + 2R/2R) ®	29 (55.8%)	27 (56.2%)		
3R/3R ^d^	23 (44.2%)	21 (43.8%)	0.961	1.020 (0.463–2.248)
2R allele ®	35 (33.7%)	37 (38.5%)		
3R allele ^e^	69 (66.3%)	59 (61.5%)	0.472	1.236 (0.693–2.204)
*ATIC* C347G (rs2372536)				
CC®	27 (51.9%)	25 (52.1%)		
GG ^a^	4 (7.7%)	7 (14.6%)	0.353	0.529 (0.138–2.028)
CG ^b^	21 (40.4%)	16 (33.3%)	0.652	1.215 (0.521–2.837)
GG + CG ^c^	25 (48.1%)	23 (47.9%)	0.987	1.006 (0.459–2.207)
CG + CC ®	48 (92.3%)	41 (85.4%)		
GG ^d^	4 (7.7%)	7 (14.6%)	0.279	0.488 (0.133–1.786)
C allele ®	75 (72.1%)	66 (68.8%)		
G allele ^e^	29 (27.9%)	30 (31.2)	0.602	0.851 (0.463–1.563)

Results are expressed in *n* (%)

*OR* odds ratio, ® reference group, *CI* confidence interval, *LL* lower limit, *UL* upper limit, *a* homozygous genetic model, *b* heterozygous genetic model, *c* dominant genetic model, *d* recessive genetic model, *e* allele model, *p p*-value for univariate regression analysis, *ATIC* 5-aminoimidazole-4-carboxamide ribonucleotide transformylase, *TYMS* thymidylate synthetase, *RFC1* reduced folate carrier-1

^*^p ≤ 0.05

## Discussion

Despite the patients’ compliance with treatment in the current research, only 41% of them exhibited a good response to MTX monotherapy at a median concentration of 25 mg/week, while 59% showed a poor response at the same median dose. In an observational study by Sergeant et al. [17], 43% of their cohort were classified as non-responders. Their baseline determinants of non-response in a multivariable logistic regression model were lower disease activity, negative RF, and a higher HAQ score. In this study, none of the different clinical elements revealed significance in the univariate regression analysis, and no clinical criteria could adequately explain or predict therapy response. So consideration of genetic factors affecting therapy response was more pertinent.

According to the current research, variations in MTX effectiveness and toxicity may be associated with MTX pathway dysregulation. Because multiple enzymes are involved in MTX metabolism, changes in enzyme accessibility and functioning may have a direct impact on MTX therapy [18].

Using the recessive genetic model, there was a significant difference in the RFC1 G80A (rs1051266) genotype distribution between non-responders and responders. This clarified that the 80 AA genotype of *RFC1* G80A was linked to a better response to MTX treatment. Multivariate regression analysis confirmed this result. It showed that *RFC1* (GA + GG) increased the risk of not responding to MTX by 3.838-fold.

RFC1, which has a high affinity for reduced folates and different hydrophilic antifolates like MTX, controls the active movement of MTX into the gastrointestinal system. It has been proposed that the *RFC1* G80A variation in exon 2 is linked to the effectiveness of MTX. It causes the replacement of histidine for arginine at amino acid position codon 27 in the first transmembrane domain of the RFC1 protein [19]. A previous study done in silico showed that this variant changed the topology of RFC1 at the S1, S2, S4, S8, S9, and S10 transmembrane domains. The S2 domain is shorter in the mutant protein than in the wild protein by three amino acid residues, while the difference in other domains corresponds to one amino acid residue. This alters how tetrahydrofolate (THF), 5-methyl-THF, and MTX are transported across the membrane [20]. It was previously shown that *RFC1* mRNA, which is affected by gene variants, has been found to affect the beneficial effects of MTX in RA [21].

In agreement with the current result, Baslund et al. [22] found that individuals with the A variant (80AA and 80GA) had higher levels of MTX uptake in antigen-stimulated CD4 + T cells and B cells than those exhibiting the 80GG variant, which may have contributed to an increase in MTX influx. Both cells are crucial in the pathogenesis of RA. Similarly, a study on Indians demonstrated that those with the *RFC1* 80 AA genotype responded to MTX better than people with the *RFC1* 80 GG genotype [23]. Furthermore, Dervieux et al. reported that *RFC1* 80AA homozygosity was linked to 3.4-fold greater amounts of MTX-PG3-5 and/or MTX-PG5 in red blood cells [24]. Also, Naushad et al. [25] showed that the *RFC1* 80A-allele increased the usefulness of MTX therapy by 1.53-fold in their meta-analysis, which included 18 studies and was representative of 3592 RA individuals, and the 80AA-genotype raised the impact by 1.85-fold with an increased MTX dose equivalent to 15 mg/week, which is comparable to the average dose in the current study. Another study demonstrated that cases with the *RFC1* 80AA genotype had a 3.32-fold greater chance of remission of RA manifestations than those with the GG genotype [26].

On the other hand, Yamamoto’s study [8], which went against the current findings, showed that there was no link between the intracellular MTXPG3-5/1–2 ratios and the *RFC-1* G80A polymorphism. Earlier studies also found no differences between the *RFC-1* gene polymorphisms and the effectiveness of MTX therapy in Chinese and European cohorts of RA patients [7, 27]. Although the outcomes of the present study illustrated that *RFC-1* G80A may be an important pharmacogenetic indicator of MTX treatment, more studies in different populations with a larger sample size are highly recommended.

TYMS is an enzyme implemented in DNA synthesis and repair. Its inhibition by MTX-PG contributes to MTX anti-proliferative and anti-inflammatory properties. The genetic variation in the TYMS gene, which involves a tandem repeat of a 28-base pair sequence in the promoter region, resulting in a 2R or 3R allele, may affect the transcriptional activity of the gene [1]. However, its potential consequences on enzyme function are still a matter of controversy, prompting a search approach to better understand the influence of this variation on pharmacogenetics. In the current study, a significant difference was found among the non-responders and responders in the dominant and codominant genetic models of *TYMS* 2R/3R, indicating that the presence of 2R/3R or 3R/3R genotypes makes them more vulnerable to a good response. The multivariate regression analysis confirmed the role of *TYMS* (rs34743033) (2R/2R) by 6.39-fold as a risk factor for non-response to MTX.

Subjects with the TYMS 3R repeat sequence exhibited higher translational efficiency than those with the TYMS 2R repeat sequence. The increased expression of the 3R allele can boost the conversion of dUMP to dTMP, reducing the amount of uracil that could get integrated into DNA and cause double strand breaks [28].

In agreement with the current result, James et al. [29] revealed that patients with the TYMS 3R3R genotype reacted more positively to therapy in 98 early RA patients. On the contrary, Lima et al. [30] stated that the TYMS 3R allele favored non-response to MTX medication in Portuguese Caucasian RA people. Although there were significant univariate analysis outcomes in their study, the multivariate test was not capable of supporting this result. Also, Muralidharan et al. [31] found that the 3R allele was greater in non-responders than in persons having remission and concluded that the TYMS 3R allele could provoke non-response to MTX in South Indian Tamil ethnicity. Other study [27] assumed that the TYMS 28-bp tandem repeat variation did not have any function in anticipating the MTX therapy outcome. Despite the promise of our findings, important study constraints, such as the relatively small population size and the presence of additional TYMS variations that may influence TS expression or activity and necessitate further research, may help explain these disparities in the results.

Besides the TYMS, MTX-PGs target the ATIC enzyme, which prompts the last two steps of de novo purine production, and raise the intracellular concentration of AICAR, which contributes to the activation of the adenosine signaling pathway. Strong anti-inflammatory functions are produced by the release of adenosine, which reduces neutrophil adhesion and suppresses the function of natural killer cells, monocytes, macrophages, and T lymphocytes [32]. The *ATIC* C347G (rs2372536) variant on exon 5 is an *ATIC* variation that changes the amino acid threonine to the amino acid serine at position 116 of the gene. The current study failed to find strong evidence linking the *ATIC* C347G variation to medication response in any of the genetic models investigated. This finding was previously reported in other research. Sha et al. [18] evaluated the ATIC C347G polymorphism in 647 RA patients and found that this variation had no effect on MTX efficacy based on both the chi-square test and binary logistic regression. The stratification of RA patients by self-reported ancestry, however, showed that Malay RA patients with minor allele G of ATIC responded better to MTX therapy than Chinese and Indian RA patients. Additionally, Sharma and Muralidharan [33, 34] found no correlation between MTX responsiveness in Indian people and ATIC C347G in their investigations.

However, a link between the *ATIC* 347 GG + GC genotype and non-response to MTX treatment was discovered in the Caucasian population by a meta-analysis based on five experiments with 1056 RA people. Another meta-analysis presented a significant link among the frequency of the *ATIC* C347G allele and the state of the MTX response in both dominant and codominant models [32, 35]. A cross-sectional investigation of 108 RA cases by Dervieux et al. [15] found that individuals with a homozygous GG of *ATIC* C347G could possess a greater percentage of excellent reactions to MTX than those with a CC or CG genotype. Kurzawski et al. [36] came to identical findings in RA patients from a Caucasian population.

The differences across the studies might be attributed to the varying selection criteria used for the studied individuals, their varied ethnicities, geographic locations, concurrent environmental risk factors, and interactions between genes and their environment.

MTX medication has been discontinued in around 30% of patients owing to adverse effects, according to estimates. In the current investigation, the genotypes and allele frequencies of the selected variations did not show any significant associations with the deleterious effects across all genetic models.

According to Wang et al.’s findings [37], *RFC1* G80A gene polymorphisms were not connected to MTX toxicity in Chinese Han individuals, which lines up with our results. Muralidharan et al. reported the same results in the Indian population [31] and regardless of ethnicity, the meta-analysis of Huang et al. [38] which comprised seven studies and came to the same conclusions.

The relationships between the chosen variants and MTX toxicity, however, varied among researchers. People with the *RFC1* 80AA genotype were shown to possess a greater prevalence of MTX-related hepatotoxicity and alopecia, while the TYMS 3R3R genotype was linked to a greater danger of bone marrow toxicity. Instead, Chaabane et al. [39] demonstrated that the TYMS 2R/3R repeat variant had a protective impact as MTX toxicity developed. Furthermore, Grabar et al. [40] recorded that ATIC 347G allele carriers possess a 2.5-fold increased risk of MTX-induced toxicity compared to non-carriers.

The results’ variability may be caused by changes in clinical characteristics, discrepancies in how treatment outcomes are measured, inter-ethnic differences, and an insufficient sample size. To better recognize the function of these and other genetic variations in methotrexate response and to identify patient subgroups who are more likely to experience methotrexate failure and may benefit from alternative therapies, additional research in a larger, prospectively gathered cohort with well-defined outcomes and clinical measures will be necessary. These results need to be verified by other research, and if they are, they may emphasize the need for tailored treatment for RA patients.

## Conclusion

In our RA cohort, the 80AA genotype of *RFC-*1 G80A and the 2R/3R or 3R/3R genotypes of *TYMS* 2R/3R were more vulnerable to a good response to MTX treatment. Furthermore, no clinical variables could be linked with a poor response to MTX treatment.

## Data Availability

This paper includes all data created or analyzed during this investigation.

## Abbreviations

*ACR*: American College of Rheumatology
*ALT*: Alanine aminotransferase
*Anti-CCP*: Anti-cyclic citrullinated protein antibody
*AST*: Aspartate aminotransferase
*BMI*: Body mass index
*CBC*: Complete blood count
*CRP*: C-reactive protein
*DAS*: Disease activity score
*EDTA*: Ethylenediaminetetraacetic acid
*eGFR*: Estimated glomerular filtration rate
*EULAR*: European league against rheumatism
*ESR*: Erythrocyte sedimentation rate
*HAQ*: Health assessment questionnaire
*MTX*: Methotrexate
*NSAIDs*: Nonsteroidal anti-inflammatory drugs
*PCR–RFLP *: Polymerase chain reaction-restriction fragment length polymorphism
*RA*: Rheumatoid arthritis
*RF*: Rheumatoid factor
